# A hospital-based surveillance for Japanese encephalitis in Bali, Indonesia

**DOI:** 10.1186/1741-7015-4-8

**Published:** 2006-04-07

**Authors:** Komang Kari, Wei Liu, Kompiang Gautama, Mammen P Mammen, John D Clemens, Ananda Nisalak, Ketut Subrata, Hyei Kyung Kim, Zhi-Yi Xu

**Affiliations:** 1 Department of Pediatrics, Udayana University School of Medicine, Bali, Indonesia; 2 Department of Pediatrics, Sanglah Hospital, Bali, Indonesia; 3 International Vaccine Institute, Seoul, Korea; 4 Department of Virology, Armed Forces Research Institute of Medical Sciences (AFRIMS), Bangkok, Thailand; 5 Center for Disease Control and Prevention, Bali, Indonesia

## Abstract

**Background:**

Japanese encephalitis (JE) is presumed to be endemic throughout Asia, yet only a few cases have been reported in tropical Asian countries such as Indonesia, Malaysia and the Philippines. To estimate the true disease burden due to JE in this region, we conducted a prospective, hospital-based surveillance with a catchment population of 599,120 children less than 12 years of age in Bali, Indonesia, from July 2001 through December 2003.

**Methods:**

Balinese children presenting to any health care facility with acute viral encephalitis or aseptic meningitis were enrolled. A "confirmed" diagnosis of JE required the detection of JE virus (JEV)-specific IgM in cerebrospinal fluid, whereas a diagnosis of "probable JE" was assigned to those cases in which JEV-specific IgM was detected only in serum.

**Results:**

In all, 86 confirmed and 4 probable JE cases were identified. The annualized JE incidence rate was 7.1 and adjusted to 8.2 per 100,000 for children less than 10 years of age over the 2.5 consecutive years of study. Only one JE case was found among 96,920 children 10–11 years old (0.4 per 100,000). Nine children (10%) died and 33 (37%) of the survivors had neurological sequelae at discharge. JEV was transmitted in Bali year-round with 70% of cases in the rainy season.

**Conclusion:**

JE incidence and case-fatality rates in Bali were comparable to those of other JE-endemic countries of Asia. Our findings contradict the common wisdom that JE is rare in tropical Asia. Hence, the geographical range of endemic JE is broader than previously described. The results of the study support the need to introduce JE vaccination into Bali.

## Background

Japanese encephalitis (JE), a mosquito-borne viral infection, has a high case-fatality rate (10%–30%) [1,2] with 30%–50% of survivors left with long-term neurological disability [3,4]. Some 35,000 to 50,000 cases of JE are reported annually [1-4], a likely underestimate given that JE is not a notifiable disease in most Asian countries. Following the successful control of poliomyelitis, JE has become a major cause of neurological disability among Asian children.

*Culex tritaeniorhynchus*, the major mosquito vector of JE virus (JEV), breeds by laying eggs in wet rice fields [1]. JEV is transmitted by mosquito vectors to a variety of mammals and birds [1,5]. Pigs are the most efficient amplifying hosts for dissemination of JEV, capable of exhibiting up to 9 logs of viremia [6,7]. Thus, rural areas with both rice fields and pigpens provide the best habitats for propagation and transmission of JEV. Children remain the main victims of the disease [2,8]. Most JE infections are asymptomatic, and the ratio of symptomatic to asymptomatic infections ranges from 1 in 300 to 1 in 1,000 [9-12].

JE incidence declined dramatically in Japan, Korea, China and Thailand after JE vaccine was mandated in routine childhood immunization programs [13]. Most other Asian countries have not implemented JE vaccination programs, owing partly to inadequate documentation of JE risk. For example, in Indonesia, Malaysia and the Philippines, only 284 JE cases were reported between 1986 and 1996, compared with thousands of cases during the same period in the neighboring countries of Thailand and Vietnam, which share similar natural ecological conditions [14]. We believed the low JE incidence in Malaysia, Indonesia and the Philippines probably reflected an underestimate due to lack of JE surveillance activities and diagnostic capabilities. To determine the potential high risk of JE in this region, we conducted a prospective hospital-based surveillance in Bali, Indonesia, from July 2001 through December 2003.

## Methods

### Study area

Bali, an island located 8–9 degrees south of the equator, has mean monthly temperatures of 20–35°C and a rainy season that extends from November through April. Of approximately 3 million residents, 90% are Hindu farmers. They raise pigs and grow rice year-round, providing persistent breeding sites for JE mosquito vectors. Health care is affordable and accessible by most families. The island is divided into nine administrative units (8 districts and 1 town), each equipped with a government-sponsored modern hospital, several sub-district health centers and many village health clinics. In addition, 14 private clinics provide pediatric outpatient care; inpatient services, however, are provided by 10 government hospitals (1 military).

### JE surveillance system

In July 2001, we established a case referral system that included all health care facilities on the island providing care for children under 12 years of age (599,120 children). The pediatric inpatient departments of the 10 government-sponsored provincial and district hospitals were used as surveillance centers. Patients suspected of JE were referred from other health care facilities to 1 of the 10 surveillance centers for further evaluation and treatment. All pediatricians who work on infectious diseases from the 10 surveillance centers and a subset of general practitioners from sub-district health centers attended workshops designed to increase awareness of JE and to define the safe handling of patients and specimens; they were also encouraged to report and refer suspected JE cases. Television programs and newspapers encouraged parents to bring children with signs or symptoms suggestive of JE to surveillance centers for treatment.

### Enrollment criteria

Children suspected of JE were eligible for this study if they were Bali residents under 12 years of age, and were admitted or referred to one of the pediatric departments of the ten surveillance centers. A suspected case was defined as a child who experienced acute onset of fever (body temperature = 38°C, axillary) with any neurological deficits (e.g., seizure, cranial nerve or sensory deficits, abnormal movements, weakness of one or more limbs) and/or changes in mental status (e.g., confusion, disorientation or coma) and/or signs of meningeal irritation (e.g., headache, vomiting, neck stiffness, Kernig's Sign). Exclusion criteria included confirmed bacterial meningitis, cerebral malaria and tumors causing similar neurological deficits.

### Clinical evaluations and specimen collection

The institutional review boards of the Udayana University in Bali, the International Vaccine Institute (IVI) in Seoul, and the Program for Appropriate Technology for Health (PATH) in Seattle reviewed and approved the study documents. Written informed consent was obtained from parents or guardians prior to enrollment of their children in the study. Clinical symptoms on admission and upon discharge were captured on case report forms. Venous blood and cerebrospinal fluid (CSF) were collected on admission and whenever possible upon follow-up, usually 1–2 weeks later. Initial laboratory evaluations aimed to rule out treatable meningitis with a bacterial etiology. Specimens were stored at -70°C for further tests.

### Confirmatory diagnosis of JE

The laboratory protocol of JE diagnosis was based on detection of anti-JEV IgM in CSF or serum by the enzyme-linked immunosorbent assay (MAC ELISA) as described by Burke and colleagues [15,16]; test reagents were provided by the Armed Forces Research Institute of Medical Sciences (AFRIMS) in Bangkok. A subject was diagnosed with "confirmed JE" if anti-JEV IgM was present in the CSF (1:10 dilution). If the virus-specific IgM was detected only in serum (1:100 dilution), the diagnosis of "probable JE" was considered. Because dengue virus, another flavivirus, co-circulates with JEV within the study area, an IgM capture ELISA for dengue was concurrently performed on all specimens [17].

### Statistical methods

The population was projected for 2001–2003 on the basis of a 2000 census. We used a Poisson model to estimate the effects of age intervals on incidence rates [18]. Data cleaning and verification programs and final data analyses were completed using the STATA/SE 8 and Microsoft Access (version 2002) software programs.

## Results

### Enrollment and verification of JE patients

From July 2001 through December 2003, 239 eligible children were enrolled. Of these, 140 (59%) directly visited one of the ten surveillance centers, 27 (11%) and 43 (18%) were referred from sub-district health centers and village health clinics, respectively, and 29 (12%) were referred from private clinics.

Admission CSF and serum specimens were obtained from all enrolled children, but convalescent CSF samples were only available for 104 patients. Of the 239 enrollees, 86 (36%) were classified as "confirmed JE" (CSF detection), 4 (2%) as "probable JE" (only serum detection) and 149 (62%) as "non-JE encephalitis." No anti-dengue IgM antibodies were detected in CSF or serum specimens.

### JE incidence and case-fatality rates

Among the 86 confirmed and 4 probable JE patients, only 1 was over 10 years. Considering the timing of the remaining 89 JE cases, the attack rate for the first 6 months of surveillance (July to December 2001) was similar to the subsequent two calendar years, with an average annual detection of 36 cases. Thus, the annualized JE incidence per 100,000 children less than 10 years of age in Bali was 7.1 in 2001, 7.0 in 2002, and 7.1 in 2003, respectively, with an average of 7.1 (95% confidence interval [CI], 3.6–10.5 per 100,000). Seventy percent of the cases were younger than 5 years, giving an annualized JE incidence of 9.1 per 100,000 (95% CI, 5.6–12.7). The rates were 4.6 (95% CI, 1.8–7.4) and 0.4 (95% CI, 0–1.7) for the age groups of 5–9 and 10–11 years, respectively (Table 1). The male-to-female ratio was 1.7:1 (P < 0.05); however, the ratio was 1:1.2 among cases <12 months old.

### Potential impact of incomplete availability of second CSF specimen

A past study suggests that anti-JEV IgM levels in CSF usually peak during the 2^nd ^to 4^th ^week of illness, and only 75% of confirmed JE cases have detectable IgM antibodies in CSF specimens during the first week of illness [19]. In our study, of the 135 patients with admission-only CSF samples collected during the first week of illness, 32 (24%) exhibited positive anti-JEV IgM. Of the 104 enrollees with convalescence CSF samples collected at least 1 week after illness onset, 54 (52%) tested positive for anti-JEV IgM. The positivity rate for the paired CSF group was significantly higher than for the acute phase-only CSF group (χ^2 ^= 20.31, P < 0.001). Among the 54 confirmed JE cases with paired CSF samples, 38 (70%) demonstrated anti-JEV IgM in both specimens. The remaining 16 (30%) cases had negative first samples but positive convalescence samples. Therefore, in addition to the 32 (70%) confirmed JE cases in the acute phase-only CSF group, 14 (30%) more JE cases would have been detected if two sequential CSF samples had been collected for this group.

### Discharge status of children infected with JE

Nine (11%) of the 86 children with confirmed JE died during hospitalization. Of the survivors, 31 (36%) had symptomatic neurological sequelae at discharge that included muscular paralysis, increased tonus, ataxia, epilepsy, clonus, chorea and respiratory distress. Of the 4 probable JE cases, 2 were fully recovered, 1 had aphasia and limb paralysis, and 1 had aphasia and extra-pyramidal symptoms (Table 2).

### Distribution of JE cases by month

**Figure 1:**
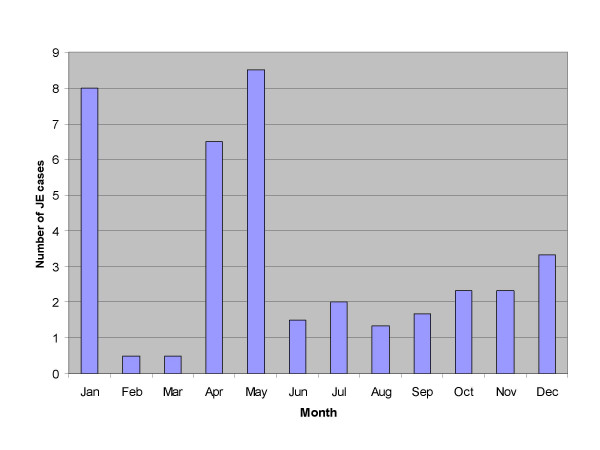
Average monthly hospital admission of children with Japanese encephalitis (JE) in Bali, Indonesia, July 2001 through December 2003

As shown in Figure 1, no calendar month consistently lacked reports of JE cases throughout the 2.5-year study period, though several calendar months showed at least one JE case in one year but not in the other. For instance, JE cases were reported during February and March in 2002, but not in 2003; no case was reported in September and October of 2001, but 4 and 8 cases were reported in these 2 calendar months in 2002 and 2003 respectively. Hence, unlike the strict summer seasonality in temperate JE-endemic countries [1,2], JEV appears to be transmitted year-round in Bali. Seventy percent of JE cases were detected between December and May, a typical 1-month lag behind the usual rainy season (November-April), which is required for JEV incubation and dissemination.

### Geographical distribution of JE

JE occurred in all nine administrative districts of Bali. The incidence was higher in the southern plains, where rice fields are more widely distributed, than in the mountainous districts of northern Bali.

Only one village produced more than a single JE case during the course of the study. The interval between the onset of illness for the 2 cases was 2.5 months, five times longer than the average 2-week incubation period of the disease, suggesting that these cases were unrelated.

## Discussion

Tropical countries of eastern Asia (Indonesia, Malaysia and the Philippines) have conventionally been considered to have low JE risk [2]. In Indonesia, for example, only 41 JE cases were reported for the whole country during the 11 years from 1986 through 1996 [14]. Although JEV was isolated from the mosquito vector in 1972 and serological surveys confirmed the prevalence of JEV infections in endemic wild birds and domestic animals, only sporadic human JE cases have been reported in Indonesia [20-22]. Previously, this low JE-risk to humans was attributed to a less virulent JEV circulating in the region [23]. Our study results suggest the contrary. In Bali alone we identified 90 JE cases in humans within just 2.5 years. Even this may be an underestimate given that 135 of the 239 suspected JE cases provided only acute-phase CSF samples within the 1^st ^week of illness onset, of which only 32 JE cases were detected. If we assume that 30% of JE cases were undiagnosed in the acute-phase-only CSF group, approximately 14 additional JE cases would have been detected if a convalescence CSF sample had been available, making the total confirmed and probable JE cases 104. The annualized JE incidence rate among children under 10 years of age could then be adjusted from 7.1 to 8.2 per 100,000. Thus, the data obtained through comprehensive surveillance indicate that JEV is hyperendemic in Bali, that it causes substantial human illness, and that it circulates year-round. Our findings in a tropical country that was presumed to have low risk of JE may necessitate more intensive surveillance to assess JE-risk fully in other countries of tropical Asia.

Most evaluations of JE disease burden have been based on clinical reports of acute viral encephalitis without laboratory confirmation. Accurate estimation of age-specific incidence of JE is rare. The pre-adjusted annual incidence rates of 9.1 per 100,000 in children 0–4 years of age and 7.1 per 100,000 in children 0–9 years in Bali are similar to the incidence rates of 7–11 per 100,000 among children aged 0–9 years living in high-risk provinces of northern Thailand and 6.0 per 100,000 in children aged 0–9 years in endemic areas of Taiwan [3,24,25]. Age-specific incidence rates in placebo groups observed during vaccine trials in Asian countries have fluctuated between 15 and 25 per 100,000 [14]. However, the study populations for vaccine trials are selected from high-risk areas, and JE incidence in such studies is measured more rigorously using advanced technologies.

JE in temperate and subtropical Asian countries (China, Japan, Korea, Nepal, northern Vietnam and northern Thailand) is characterized by cyclic epidemics and JE outbreaks every 5–15 years [26]. During the inter-epidemic years, JE incidence rates significantly decline [2,4]. In contrast, in Bali, we observed consistent JE incidence rates of about 7.1 per 100,000 over 2.5 consecutive years. Further studies are needed to assess the validity of this finding over longer periods of time and to determine whether cyclic epidemics occur in Bali. In temperate JE-epidemic regions, a strict summer seasonality of JE has been observed; the mechanism of over-wintering of the virus in the winter and spring is unclear [1,4,5]. In Bali, where JEV appears to circulate year-round, this mechanism may not apply.

In the northern regions of Thailand and Vietnam, the highest JE attack rates are in children aged 5–9 years [27-29]. In Bali, however, 70% of the JE cases were children under 5 years of age (Table 1). This suggests a greater intensity of transmission that exposes children to JEV at a younger age. Similar to findings elsewhere, our study suggests that amongst children above the age of 1 year, males have a greater predilection to JE than females. This may be related to boys engaging in more outdoor activities than girls, leading to greater exposure to JE-infected mosquitoes [3,4]. Also consistent with other studies, no clustering of JE cases was evident [3,4]. Though emergency yellow fever immunization in response to yellow fever outbreaks is effective in ameliorating the person-to-person transmission involving domicile mosquitoes [30], such a strategy of emergent JE vaccination in response to identification of an initial JE case would be ineffective.

The transmission cycle of JEV relies solely on the mosquito vector and the amplifying non-human host (primarily the pig). Humans are accidental hosts and are not required for virus transmission. Bali represents an interesting, but not necessarily unique, ecological setting wherein both pig raising and rice farming take place year-round, representing ideal conditions for consistent JEV propagation. As Balinese are predominantly Hindu (unlike other Indonesians, who are predominantly Muslim), pork is a major food item with a pig-to-human population ratio of approximately 1:2. We found that 280 (70%) of 400 pigs in Bali showed hemagglutination inhibition antibody against JEV (unpublished data). Consequently, pigs probably serve as the primary amplifying hosts of JEV in Bali. Rice cultivation in Bali is not seasonal. Therefore, only parts of rice fields are filled with water at any given time and they are periodically drained as part of intermittent irrigation practice [31]. As a result, the rainy season appears to facilitate water filling and mosquito breeding within the rice fields. Hence, the tropical climate and agricultural practices in Bali work in concert to accelerate the year-round transmission of JEV via the animal-mosquito cycle.

We note certain study limitations. First, JE infections were found in children presenting with acute flaccid paralysis (AFP) in southern Vietnam [32]. In retrospect, we found that we failed to include 14 AFP cases documented within Bali whose ages were within the case definition of our surveillance. Second, our surveillance approach targeted children seeking medical attention and missed JE cases that may have led to early pre-hospital deaths. Likewise, mild JE cases manifesting only by febrile convulsions may have been missed. These omissions may have contributed further to the underestimation of true JE incidence in our study.

## Conclusion

Given the particular social and ecological setting, the data obtained from Bali may or may not be applicable to the rest of Indonesia. However, our findings clearly suggest that countries previously classified as at low JE risk may be so only because of insufficient surveillance data. Additional studies are warranted in areas presumed to have low JE risk.

Current JE vaccines are safe, effective and cost-effective [33-35]. By any standard, JE is a major public health problem in Bali and will remain so given that humans are accidental (not required) hosts in the vector-animal (pig) transmission cycle. Effective JE vaccination programs provide the only solution to the ongoing threat of JE to endemic populations such as those on Bali.

## Competing interests

The author(s) declare that they have no competing interests.

## Authors' contributions

Dr. Komang Kari and Dr. Kompiang Gautama established and monitored the surveillance activities, and enrolled and evaluated the patients. Dr. Wei Liu tested specimens, collected, checked and analyzed surveillance data, and wrote and edited text for the manuscript. Dr. Mammen P. Mammen Jr. and Dr. Ananda Nisalak provided expertise on JE diagnosis and edited the manuscript. Dr. John D. Clemens provided guidance and critique during data analysis and helped with writing and editing the manuscript. Dr. Ketut Subrata and Ms. Hyei Kyung Kim contributed to data collection and data analysis. Dr. Zhi-Yi Xu designed the study, guided data collection and statistical analysis, and wrote and edited the manuscript text.

## Pre-publication history

The pre-publication history for this paper can be accessed here:



## Figures and Tables

**Table 1:** Age-sex distribution of Japanese encephalitis cases among Balinese children, July 2001 and December 2003

Age group (years)	Confirmed JE (Probable JE)			Estimated mid-2001 population	Annualized incidence† (95% CI)
			
	Male	Female	Total		
0 – 4	38 (2)	22 (1)	60 (3)	275,500	9.1 (5.6–12.7)
5 – 9	15 (1)	10 (0)	25 (1)	226,700	4.6 (1.8–7.4)
10 – 11	1 (0)	0 (0)	1 (0)	96,920	0.4 (0–1.7)

Total 0–11	54 (3)	32 (1)	86 (4)	599,120	6.0 (4.1–8.0)
† per 100,000					

**Table 2:** Discharge status of Japanese encephalitis cases in surveillance hospitals, Bali, July 2001 and December 2003

Discharge status	Confirmed JE (n = 86)	Probable JE (n = 4)	Total (n = 90)
Alive and well	43 (50%)	2 (50%)	45 (50%)
Alive but disabled	31 (36%)	2 (50%)	33 (37%)
Alive but still sick	3 (3%)	0 (0%)	3 (3%)
Dead	9 (11%)	0 (0%)	9 (10%)

Total	86 (100%)	4 (100%)	90 (100%)
